# Feasibility of label-free multiphoton luminescence imaging to track the fate of inhaled gold nanoparticles in *ex vivo* lung tissue

**DOI:** 10.1117/1.BIOS.3.4.043105

**Published:** 2026-09-29

**Authors:** Sean Burkitt, Olga Eremina, Alexander Czaja, Boyi Wang, William Wallace, Cristina Zavaleta

**Affiliations:** a University of Southern California, Department of Biomedical Engineering, Los Angeles, California, United States; b USC Michelson Center for Convergent Bioscience, Los Angeles, California, United States; c USC Norris Comprehensive Cancer Center, Keck School of Medicine, Translational Pathology Core, Department of Pathology, Los Angeles, California, United States

**Keywords:** gold nanoparticles, biodistribution, digital pathology, multiphoton imaging, multiphoton luminescence

## Abstract

**Significance:**

Understanding the biological journey of inhaled gold nanomaterials is critical for nanotoxicology and therapeutic delivery. Conventional tracking methods often require fluorescent labeling, which can alter nanoparticle (NP) surface chemistry and lead to "false-positive" signals if the label dissociates.

**Aim:**

In this feasibility study, we utilize multiphoton luminescence (MPL) imaging to track the long-term biodistribution of 50 nm AuNPs following inhalation exposure at 1 week and 1 month.

**Approach:**

Mice were administered AuNPs via intranasal inhalation in four experimental groups: low (0.1 nM), medium (1 nM), high (10 nM) doses for 1 week, and a medium dose (1 nM) for 1 month. Organs were harvested and analyzed using ICP-MS for total gold content and MPL imaging for label-free visualization of AuNP deposition.

**Results:**

AuNPs remained localized within the lungs with no evidence of systemic translocation to the liver, spleen, brain, or kidneys at either time point. MPL imaging revealed that lung concentration followed a linear scaling model at one week. MPL imaging also revealed the long-term identification and localization of AuNPs within the lung at 1 month postinhalation.

**Conclusions:**

MPL imaging is superior to brightfield microscopy for NP detection, enabling the visualization of dose-dependent aggregation and deep-tissue mapping. These findings demonstrate the feasibility of MPL imaging as a label-free complementary imaging approach for pulmonary nanoparticle biodistribution studies.

Statement of Discovery We report on the feasibility of label-free multiphoton luminescence (MPL) imaging for visualizing retained gold nanoparticles (AuNPs) in *ex vivo* lung tissue following in vivo intranasal administration. The workflow enables large-area, high-resolution, high-throughput localization of AuNPs without fluorescent labels and complements ICP-MS by preserving spatial tissue context.

## 1 Introduction

Gold nanoparticles (AuNPs) are at the forefront of biomedical and industrial innovation, driven by their distinctive optical and electronic properties.^1^^–^^5^ Current research is heavily focused on their integration into photothermal cancer therapies, nanotheranostics, targeted drug delivery, and antimicrobial treatments.^6^^–^^18^ However, the rapid adoption of these non-biodegradable materials has raised significant concerns regarding their long-term biological fate, as their toxicity and retention patterns remain poorly understood.^19^^–^^23^ This necessitates rigorous evaluation of their biodistribution and clearance over time.

The tracking of inhaled nanomaterials, in particular, is essential for assessing both environmental toxicity and the efficacy of aerosolized drug delivery systems. Over the last few decades, research into the health effects of ultrafine nanomaterials in air pollution and the toxicological impacts of engineered nanomaterials has converged. While air pollution research is long-established and linked to millions of premature deaths annually, primarily due to cardiovascular and pulmonary complications,^24^^–^^26^ nanotoxicology is a rapidly expanding field necessitated by the integration of nanotechnology into medicine and industry.

Beyond the well-documented respiratory and cardiovascular risks,^24^^,^^27^ emerging evidence suggests that NP exposure is associated with a wide array of extrapulmonary effects, including metabolic disorders, neurological diseases such as Alzheimer’s and Parkinson’s, developmental issues *in utero*, and damage to various organ systems ranging from the liver and kidneys to the skin and reproductive organs.^28^^–^^32^

Although AuNPs are widely used in biomedical research, their *ex vivo* localization is often confounded by the limitations of external labels. Labels can dissociate or change the physicochemical profile of the NP, leading to inaccurate biodistribution data.

Standard clinical pathology, which relies on hematoxylin and eosin (H&E) staining and brightfield microscopy, is largely ineffective for detecting these submicroscopic particles.^33^^–^^35^ At approximately 50 nm, AuNPs are well below the diffraction limit and lack the intrinsic contrast needed for visualization.^36^^,^^37^ Although alternative techniques such as fluorescence or radiolabeling exist, they suffer from signal degradation and marker dissociation over extended periods.^38^^–^^40^ Advanced methods also present significant trade-offs: electron microscopy offers high sensitivity but is limited by a narrow field of view and disruptive sample preparation, while inductively coupled plasma mass spectrometry (ICP-MS) provides high accuracy but requires acid digestion, which destroys the essential spatial context of the cellular landscape.^33^^,^^41^^,^^42^

Multiphoton luminescence (MPL) imaging offers a label-free alternative by leveraging the strong plasmonic properties of gold. Under 1040 nm pulsed excitation, AuNPs produce a broadband emission that can be distinguished from endogenous tissue autofluorescence with high contrast.^43^^,^^44^

Our previous studies established MPL imaging as a label-free strategy for imaging gold NPs in vascular and tissue environments.^43^^,^^44^ Here, we evaluate the feasibility of extending this approach to pulmonary NP biodistribution following intranasal administration, where whole-tissue visualization and compatibility with routine histopathology provide unique advantages. This study investigates the feasibility of using MPL to track 50 nm AuNPs postinhalation, establishing a dose-response model and characterizing long-term retention.

## 2 Methods

### 2.1 NP Fabrication and Characterization

A modified Turkevich method was used to fabricate spherical AuNPs.^45^ Prior to synthesis, all glassware was cleaned with aqua regia. Gold(III) chloride hydrate (${\mathrm{HAuCl}}_{4}\cdot xH_{2}O$, 99.995%) and trisodium citrate dihydrate ($C_{6}H_{5}{\mathrm{Na}}_{3}O_{7}\cdot 2H_{2}O$, 99.0%) were purchased from Sigma-Aldrich. Briefly, 2 mL of $30\;\mathrm{mg}\;{\mathrm{mL}}^{-1}$ ${\mathrm{HAuCl}}_{4}$ was added to 300 mL of boiling water under vigorous stirring. Then, 2.4 mL of $30\;\mathrm{mg}\;{\;\mathrm{mL}}^{-1}$ sodium citrate solution was rapidly added. The colloidal solution was stirred for an additional 10 min. The prepared AuNPs were concentrated *via* centrifugation at 1100 g for 30 min. Deionized water (Milli-Q grade, Millipore) with a resistivity of 18.2  MΩ cm was used throughout the experiment.

The resulting colloidal solution was characterized using UV-vis and NP tracking analysis (NTA) with a NanoDrop2000 (Thermo Fisher Scientific, Massachusetts, United States) and a NanoSight NS300 (Malvern Panalytical, United Kingdom), respectively. The maximal absorption band of the AuNPs was observed at 525 nm. The size and concentration were verified with NTA measurements, which indicated the size distribution of 54±6  nm. Final stock was diluted to 10 nM with deionized water. All NP concentrations reported correspond to the particle concentration in the colloidal suspension.

### 2.2 Animal Experiments

Eight-week-old female Balb/c mice (Charles River) were used for all NP biodistribution and imaging studies. All animal procedures were approved by the University’s Institutional Animal Care and Use Committee (IACUC # 21311) and conducted in accordance with established guidelines for the humane care and use of laboratory animals.

### 2.3 Experimental Design and Dosing

Mice were split into four major experimental groups. AuNPs were administered via inhalation through the nostril (20 ul each nostril via pipette tip). Group 1 was given a low dose (0.1 nM) of AuNPs. Group 2 was given a medium dose (1 nM) of AuNPs. Group 3 was given a high dose of AuNPs (10 nM). Group 4 was also given a medium dose (1 nM) of AuNPs. A separate control group was also used that received no nanoparticle injection. Group 1 to 3 were euthanized at 1 week, and Group 4 was euthanized at 1 month post AuNP administration and the organs were harvested and processed for histology (Fig. S1).

### 2.4 Tissue Collection and Analysis

The organ harvesting process began with the euthanasia of mice via cervical dislocation under deep isoflurane anesthesia, followed by the collection, rinsing, and weighing of various tissues. Samples underwent a paraffinization protocol, which involved extended fixation in 10% neutral-buffered formalin (NBF), sequential dehydration in ethanol, and clearing with xylene. These tissues were ultimately embedded in paraffin and sliced into 5  μm-thick sections for histological and immunohistochemistry (IHC) analysis.

### 2.5 Histological Staining and Mounting

To prepare the paraffin-embedded tissue for analysis, the sections first underwent deparaffinization in xylene followed by rehydration through a descending series of ethanol washes. The staining was performed at USC’s Translational Pathology Lab. The H&E protocol employed Gill’s hematoxylin to highlight cell nuclei and Eosin Y to provide a contrasting stain for the cytoplasm. Once the staining was complete, the sections were dehydrated, cleared in xylene, and permanently mounted with Cytoseal 60 to ensure they remained preserved for imaging and further analysis.

### 2.6 Transcardiac Perfusion for Lung Tissue Preservation

To reduce residual blood and improve visualization of nanoparticles within lung tissue, mice were euthanized via gentle cervical dislocation followed by bilateral thoracotomy. The thoracic cavity was rapidly exposed through removal of the skin and rib cage to access the heart. A blunted 20-gauge needle was inserted through the left ventricle into the ascending aorta and secured in place with a vascular clamp. The right ventricle was punctured to allow drainage during perfusion.

Transcardiac perfusion was performed using a syringe pump at a constant flow rate of 200  μL/s. Initially, 40 mL of saline containing 0.2% heparin was perfused to remove circulating blood, followed by 20 mL of 4% paraformaldehyde (PFA) at the same flow rate for tissue fixation. Successful perfusion was confirmed visually by increased opacity of the lungs and other organs. Following perfusion, 2 mL of saline was administered via oral gavage to reinflate the lungs prior to excision. Lung tissues were subsequently harvested and post-fixed in 4% PFA for 72 h before further histological processing.

### 2.7 Acid Digestion and Gold Content ICP-MS Analysis

Bulk gold quantification was conducted by processing organ samples through acid digestion and ICP-MS. Following the protocol established by the Nano and Pico Characterization Laboratory (NPC) at UCLA’s California NanoSystems Institute, tissue sections were weighed, placed in glass vessels, and preserved at −80°C prior to digestion. The analysis was performed using a PerkinElmer NexION 2000 ICP-MS instrument, with calibration standards created via serial dilutions of the original AuNPs injection stock in Milli-Q water to maintain measurement accuracy.

To ensure the reliability of the results, the final gold content for each sample was normalized to its specific tissue mass and reported in nanograms of gold per milligram of tissue (ng/mg). Rigorous quality control was maintained by analyzing blank samples and standard reference materials to verify reproducibility. Furthermore, each sample was processed in triplicate to confirm the precision of the gold quantification.

### 2.8 Brightfield and MPL Imaging Setup

The imaging protocol utilized a combination of brightfield and MPL microscopy to characterize tissue histology and AuNP distribution. Initially, brightfield imaging was performed with a Leica Mica system at 10× magnification, using whole-tissue stitching to capture comprehensive histological features. Subsequent MPL imaging was conducted on a Nikon A1R HD upright microscope equipped with a 25× water immersion objective and a Chameleon Discovery ultrafast tunable laser. The laser was operated at a pulsed excitation wavelength of 1040 nm with an 80 MHz repetition rate, providing an average power ($P_{\mathrm{avg}}$) of 100 mW, pulse energy ($E_{\text{pulse}}$) of 1.25 nJ, and peak power ($P_{\text{peak}}$) of 8.93 kW. To ensure thermal stability and prevent photothermal damage or deformation of the AuNPs, the laser was equilibrated for 1 h prior to use, and power levels at the sample were carefully optimized.

Emission signals were captured through a Nikon IR ND filter and separated into four descanned DU4 detectors using bandpass filters at 450/50 nm (DAPI), 525/50 nm (FITC), 600/50 nm (TRITC), and 685/70 nm (Cy5) to capture the broadband emission of AuNPs and tissue autofluorescence. To ensure precise localization, gold emission was isolated by correlating the Cy5 (650 to 720 nm) and DAPI (425 to 475 nm) channels, which effectively removed tissue contributions. Multiphoton autofluorescence was specifically used to identify regions of interest and visualize the precise localization of gold nanoparticles within the tissue sections. By acquiring tiled images across the entire sample and utilizing a software-based overlay, we were able to enhance both the localization and the quantification of the AuNP signals without inducing morphological artifacts in the tissue.

### 2.9 Data Normalization, Image Processing and Statistical Analysis

All quantitative data were normalized within experimental groups and compared across imaging modalities including MP, brightfield, and ICP-MS. Statistical analyses and additional data processing were conducted in Microsoft Excel. Where mentioned, statistical significance is given at a 95% confidence level (p<0.05). Error bars are given as ± the standard error of the mean where present. Determination of statistically significant differences was performed with the Wilcoxon signed-rank test.

## 3 Results

### 3.1 ICP-MS Confirms Localized Deposition

ICP-MS analysis established that 50 nm AuNPs remained entirely localized within the respiratory tissue. No systemic distribution was detected in the brain, liver, spleen, or kidneys at either 1 week or 1 month [Fig. 1(a)]. At the 1-week time point, the lung gold concentration scaled linearly with the administered AuNP dose [Fig. 1(b)]. ICP-MS results showed gold biodistribution across organs and dose-dependent accumulation in the lungs at one week. Although ICP-MS provides highly sensitive quantification of total tissue gold content, MPL uniquely preserves the spatial organization of nanoparticles within intact histological specimens. These techniques therefore provide complementary quantitative and spatial information for pulmonary nanotoxicology studies.

**Fig. 1:**
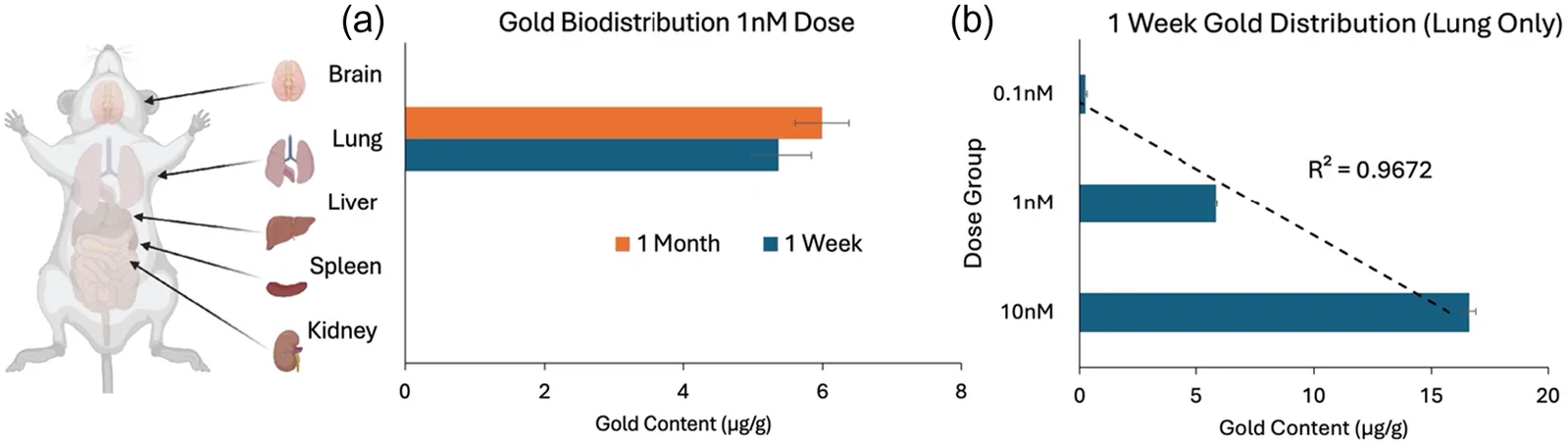
ICP-MS quantification of gold following inhalation of AuNPs at 1 week and 1 month. (a) Bulk gold biodistribution across major organs with 1 nM AuNP inhalation at 1 week and 1 month. (b) Bulk gold biodistribution in the lung only at 1 week across increasing doses of 0.1, 1, and 10 nM. Note how gold is localized exclusively to the lung at both time points. No significant difference of gold content with the lungs was observed between the 1 week and 1 month time points. Increased gold content is found within the lung across increased dosing groups (0.1, 1, and 10 nM).

### 3.2 Label-Free MPL Imaging of AuNPs

As previously reported, we chose an excitation wavelength of 1040 nm.^44^ A significant advantage of using this specific wavelength is its ability to simultaneously excite eosin and endogenous tissue reporters (SHG and autofluorescence). This allows for the concurrent visualization of tissue morphology and NP localization, providing the essential spatial context required for accurate biodistribution analysis.

Because AuNPs produce a broad emission spectrum that overlaps with tissue autofluorescence and eosin (500 to 700 nm). A four-channel emission detection workflow was implemented to isolate the various signals using standard fluorescence filters DAPI, FITC, TRITC, and Cy5. NP luminescence is most distinct in relation to tissue emission in the DAPI (425 to 475 nm) and Cy5 (650 to 720 nm) ranges (Fig. 2). By correlating these two specific channels, a binary NP localization map can be generated. This method allowed us to effectively remove background noise and significantly reduces false positives, ensuring high specificity in AuNP detection.

**Fig. 2:**
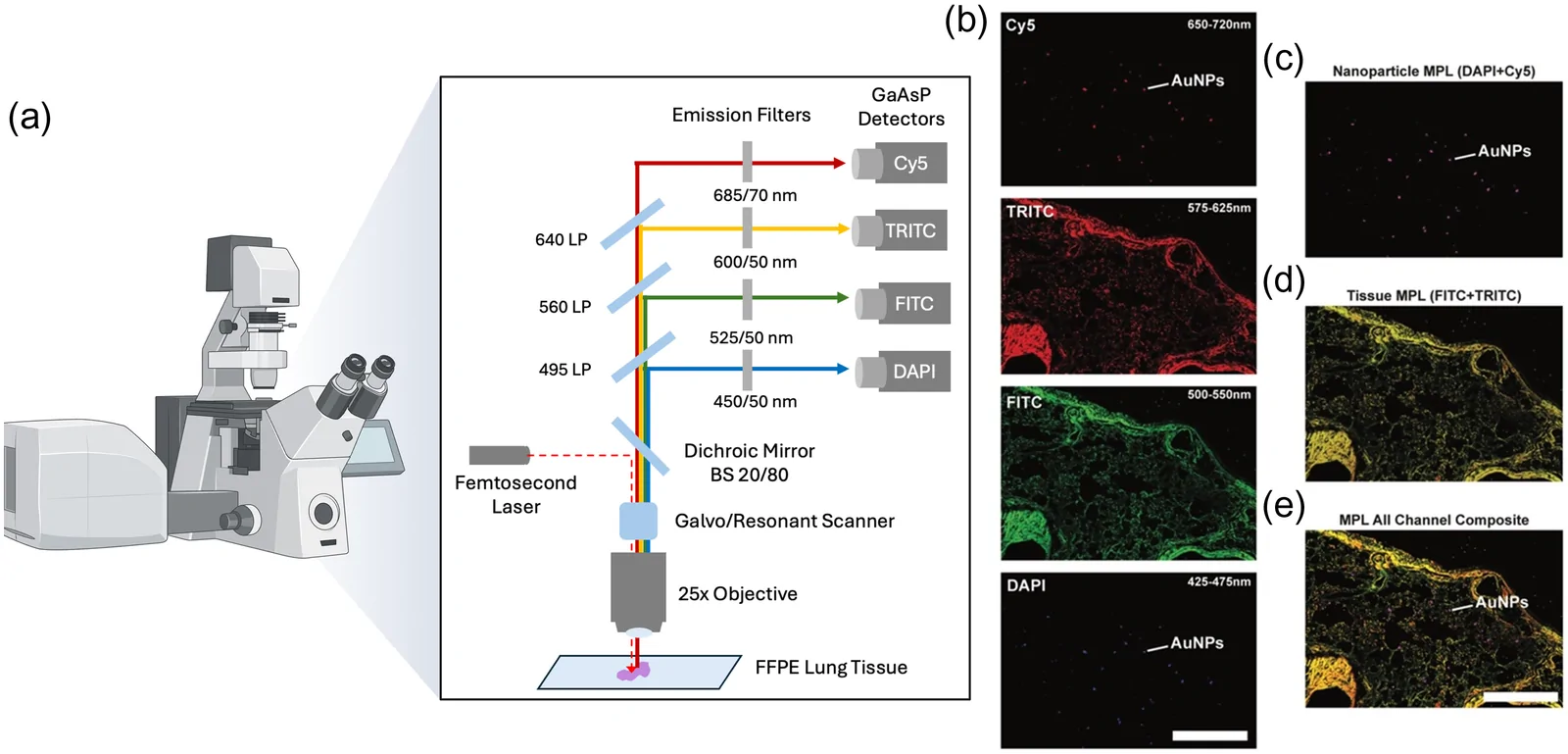
(a) Multiphoton microscope schematic. (b) Lung histology images using individual detector channels showing Cy5, TRITC, FITC, and DAPI. (c) Due to the broadband emission spectrum of gold, nanoparticles are visible in the DAPI and Cy5 channels. (d) Tissue autofluorescence is most prevalent in TRITC and FITC channels and is used for representing tissue architecture. (e) Final composite image showing tissue in yellow and AuNPs in purple. Scale bars represent 100  μm.

The primary strength of MPL imaging of AuNPs is its broadband spectral emission. Unlike standard fluorescent dyes or endogenous fluorophores, which usually emit within narrow windows of approximately 100 nm, AuNPs emit across a massive range from the UV to NIR (380 to 730 nm+). This unique property provides the flexibility to select detection channels that bypass tissue autofluorescence entirely, significantly enhancing imaging contrast. In addition, this broad emission allows for the easy integration of AuNPs with common histological stains, such as H&E and trichrome, without the need for additional labeling.^43^

The MPL microscope setup and imaging channels are detailed in Fig. 2. The broadband gold signal was successfully separated from tissue autofluorescence (eosin and second harmonic generation (SHG)) by correlating specific channels [Figs. 2(d) and 2(e)].

### 3.3 Gold NP Localization in Histology Sections

Although brightfield microscopy of FFPE histology sections paired with H&E staining is the gold standard for assessing tissue morphology, it faces significant hurdles when tasked with localizing NPs. In a comparative study, mice were administered 50 nm AuNPs at both “high” (10 nM) and “low” (0.1 nM) doses via inhalation, with lung tissues subsequently prepared using conventional H&E staining. Although the lung was clearly identified as the organ with the most gold content via ICPMS, brightfield microscopy failed to identify AuNPs in the H&E-stained histology sections (Fig. 3). Individual and small accumulations of AuNPs often fall below the diffraction limit, and their detection is further complicated by low contrast, optical scattering, and the fact that the purple and pink hues of H&E staining can effectively mask the particles.

**Fig. 3:**
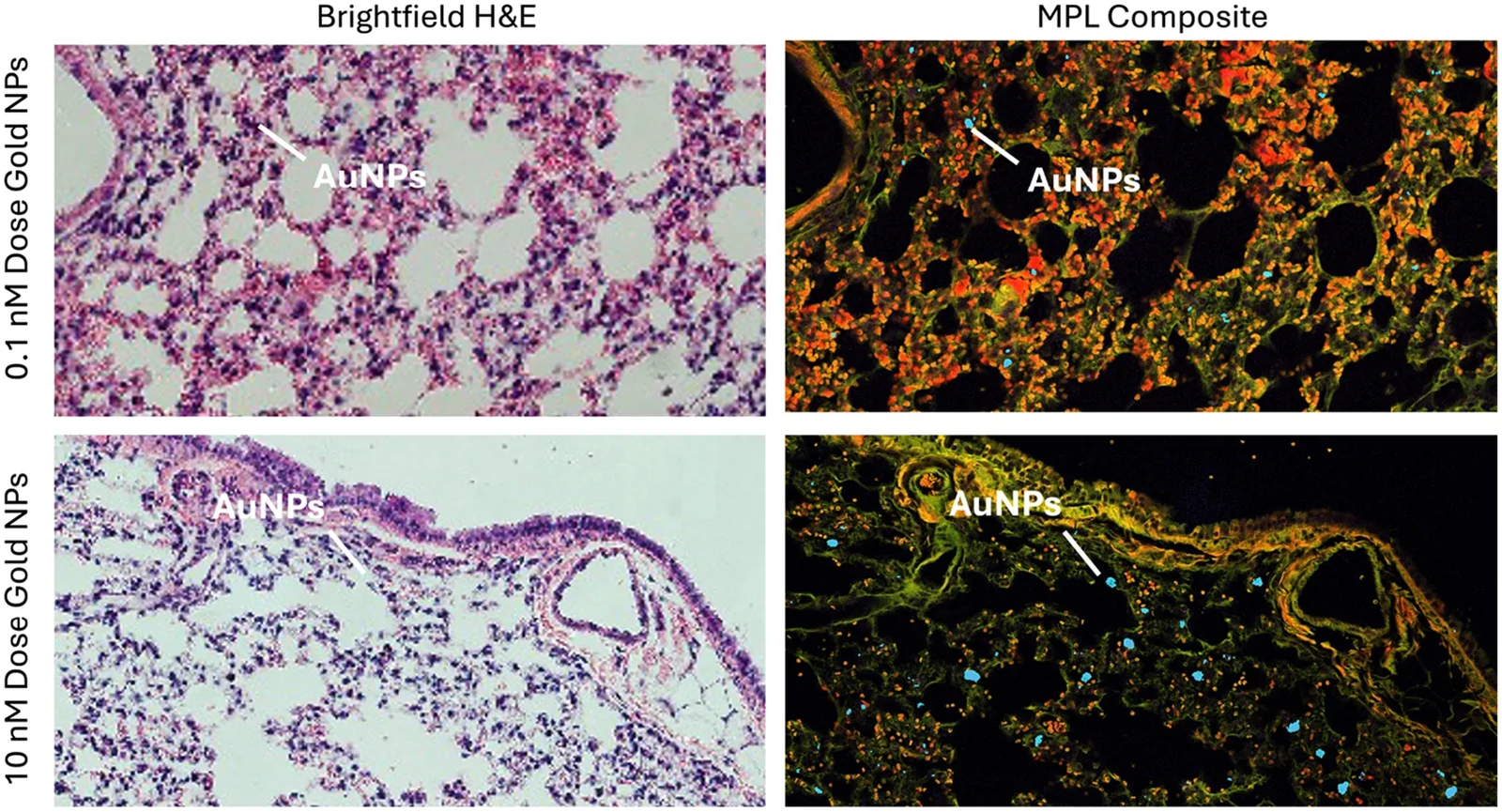
Comparison of brightfield and MPL images of FFPE H&E stained mouse lung sections. Mice were dosed with either 0.1 nM or 10 nM of AuNPs via intranasal inhalation. At 1 week postinhalation, mice were euthanized and organs were harvested. Notice how brightfield imaging is unable to identify the presence of the AuNPs across the histology section. MPL imaging clearly highlights the regions across the sample containing AuNPs. AuNPs are revealed in blue. Eosin autofluorescence and tissue autofluorescence/second harmonic generation highlight alveoli and tissue structure in yellow/green. NP emission composite from 425 to 475 nm and 650 to 720 nm highlight the presence of NP accumulation which is not present with brightfield imaging.

The visibility of these NPs is highly dose-dependent under traditional brightfield imaging. In high-dose scenarios, large NP aggregates may appear as dark black or purple features within cells. However, at lower, more physiologically relevant doses, the NPs are more diffusely distributed and remain nearly indistinguishable from the background. This scenario is a critical data gap, as pathologists have traditionally "inferred" the presence of NPs based on darkly pigmented regions. This method lacks the specificity needed to map a full biodistribution profile of these AuNPs at the cellular level.

MPL imaging is a noticeably more robust alternative that overcomes these brightfield limitations. As a label-free and non-destructive technique, MPL imaging leverages the unique intrinsic optical properties of AuNPs. It bypasses the resolution and contrast issues inherent in traditional microscopy, allowing for a much more precise and optimized workflow for tracking NPs within complex biological environments. Even diffraction-limited nanoparticles are visible via MPL imaging.^43^ MPL imaging clearly reveals AuNPs across the same H&E tissue sections imaged with brightfield microscopy (Fig. 3 is highlighted in blue).

Comparative imaging demonstrated that standard brightfield H&E imaging failed to identify the presence of AuNPs, whereas MPL clearly highlighted the AuNPs in blue against the yellow/green tissue structure (Fig. 3).

To enhance imaging quality, a post-euthanasia perfusion of the mouse lungs was performed. Although this process successfully cleared blood cells from the lung tissue, it did not interfere with the detection or localization of the AuNPs. Consequently, MPL imaging remained a highly effective tool for identifying the NPs within the stained lung tissue, as demonstrated in Fig. S3 in the Supplementary Material.

### 3.4 Large Field of View MPL Imaging

A major advantage of MPL imaging is its capacity for high-resolution yet large field of view (FOV) visualization, which allows for a comprehensive assessment of AuNP biodistribution across entire histological sections. Unlike brightfield microscopy, which often fails to identify the presence of AuNPs, especially at lower concentrations, MPL imaging clearly highlights NP localization while simultaneously preserving the structural integrity of the surrounding tissue (Fig. S2 in the Supplementary Material). In lung sections, for instance, this approach allows researchers to observe NP distribution within the context of the alveoli and overall tissue architecture, which are highlighted through eosin and tissue autofluorescence.

When compared with traditional high-resolution techniques such as electron microscopy (EM), the benefits of MPL’s large-scale imaging become even more apparent. Although EM-based methods provide high resolution, they are often constrained to very small FOVs (typically 100  μm × 100  μm) and require destructive, multiday sample preparation, including coverslip removal and carbon coating. In contrast, MPL imaging is nondestructive and requires no additional sample preparation beyond standard FFPE sectioning. This enables rapid, whole-tissue imaging in approximately 15 min, providing a much broader biological context and significantly minimizing the risk of spatial misinterpretation that can occur when viewing isolated, small-scale regions.

The scalability of MPL imaging bridges the gap between macro-level distribution and micro-level interaction. The technique supports whole-slide scanning for global biodistribution analysis while maintaining the capability for high-resolution imaging at the subcellular level. Because the process does not modify or damage the histological sections, the same samples remain compatible with existing pathology workflows, such as H&E staining or IHC. This allows for seamless correlative imaging across multiple modalities, ensuring that the spatial relationship between the NPs and the intact biological environment is fully understood.

### 3.5 Dose-Dependent Accumulation and Aggregation

Quantification of the MPL data confirmed that both the area fraction and the average size of NP aggregates increased with the administered dose (Fig. 4). Higher doses resulted in significant variations in accumulation size within the alveolar spaces.

**Fig. 4:**
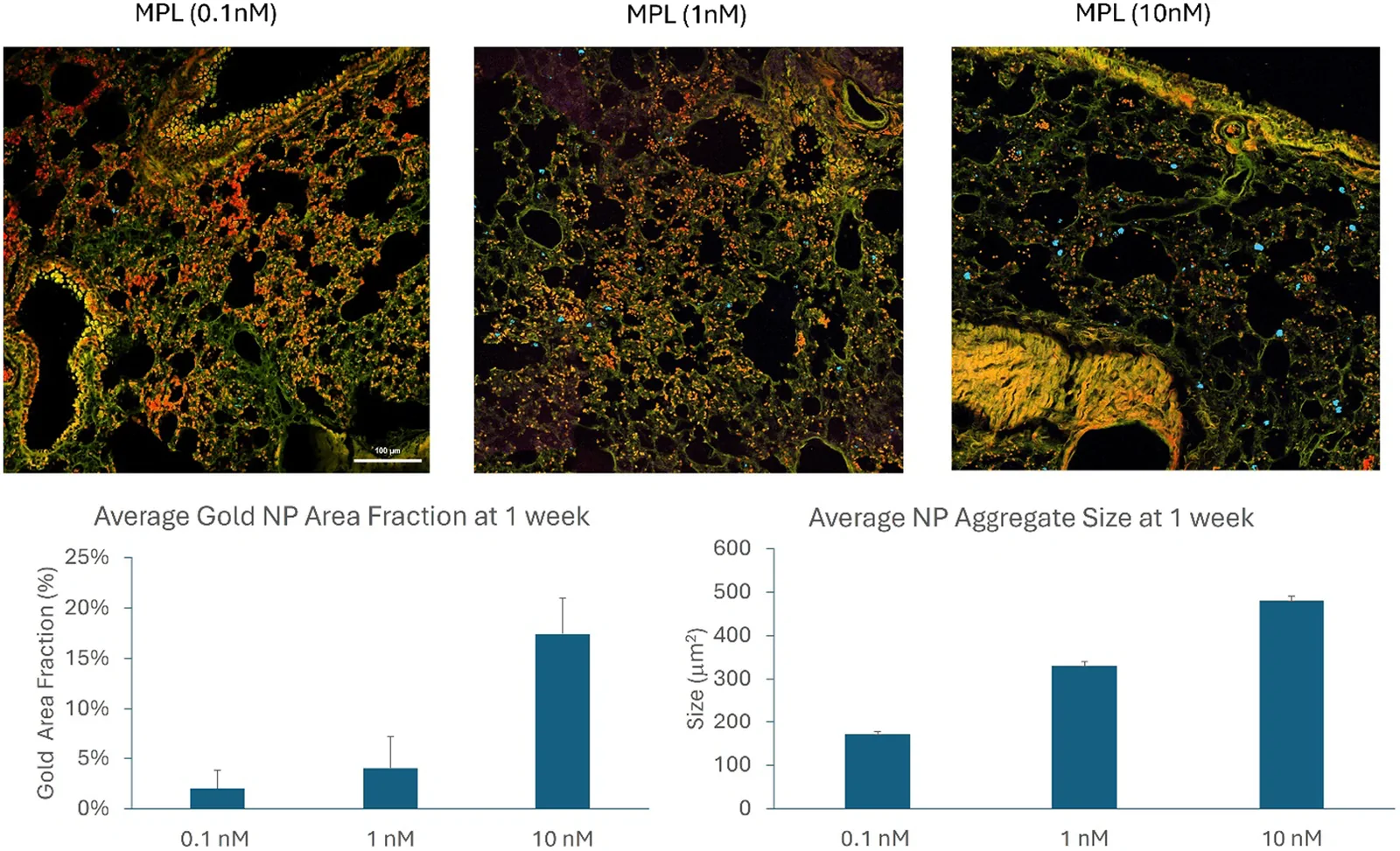
Quantitative analysis of AuNP accumulation in lung tissue at 1 week postinhalation. Fraction of gold-positive area detected in MPL images as a function of administered dose (0.1, 1, and 10 nM). Average aggregate size of AuNP signal across dosing groups.

The imaging results indicate that higher doses of AuNPs lead to the formation of larger NP aggregates within the lung (Fig. 4). Although there was no statistically significant difference in the percentage of AuNPs per MPL image area between the 0.1 nM and 1 nM doses, an upward trend in AuNP detection was observed as concentrations increased from 1 nM to 10 nM, confirming the ability of the imaging technique to capture dose-dependent increases. This initial discrepancy at lower doses may be attributed to an inconsistent distribution of AuNPs across each of the lungs following our inhalation administration to the mice. Furthermore, our analysis was limited to single tissue cross-sections rather than the entire organ, as a portion of the lung tissue was reserved for ICP-MS to validate relative gold content. Consequently, these sampling constraints may account for the variability seen in the lower-concentration groups compared with the clearer trend observed at higher doses in 1 nM and 10 nM.

Further evaluation showed that perfusing mice posteuthanasia significantly improved imaging quality by clearing red blood cells (RBCs) from the tissue, which results in a substantial reduction of autofluorescence in the TRITC red channels (Fig. S3 in the Supplementary Material). Although RBCs remained visible within the vessels of nonperfused images, this presence did not appear to interfere with the MPL detection of AuNPs (Fig. S3 in the Supplementary Material).

### 3.6 Long-Term Fate of AuNPs 1 Month Post Inhalation

MPL imaging revealed that AuNPs were still detectable in the lung at 1 month postinhalation (Fig. 5). This correlated with the ICP-MS data, which showed no significant difference in total lung gold content between 1 week and 1 month. MPL was shown to be an advantageous imaging tool to observe and monitor long-term retention of gold as it did not rely on labels that could decay or dissociate from the NPs. This opens up a whole new opportunity to study the long-term retention and tissue toxicity effects from the gold NPs residing in the lung indefinitely. The developed MPL-based workflow presents a rare opportunity to discover whether there were long-term toxicity effects like fibrosis, inflammation, or apoptosis in the exposed tissue.

**Fig. 5:**
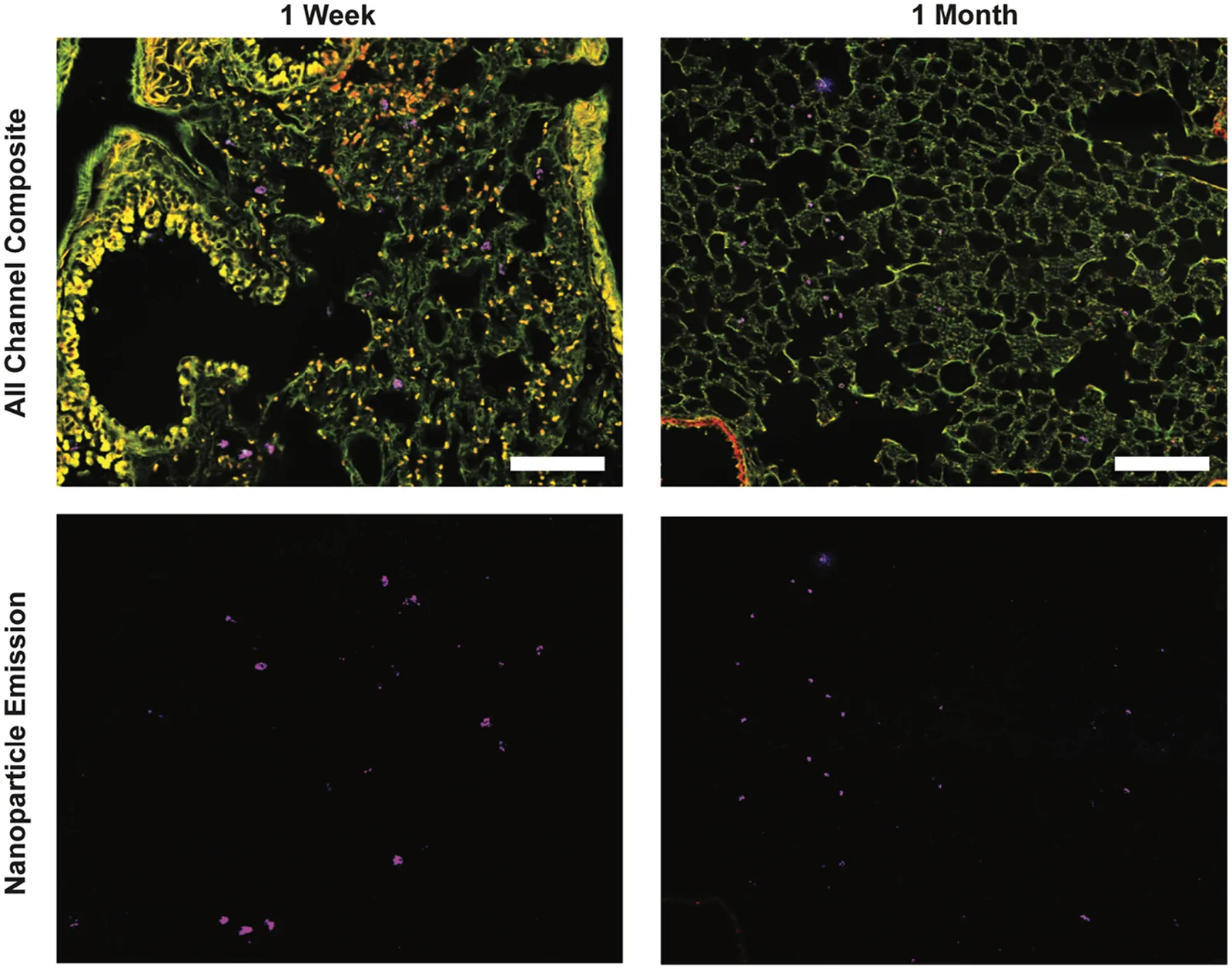
MPL imaging comparing 1-week and 1-month time points for the 1 nM dose cohort. MPL revealed that AuNPs were still residing in the lung at 1 month postinhalation. The AuNPs were clearly visible in the NP emission channel highlighted in purple. The composite image offered accurate spatial tissue detail as to where across the lung tissue the NPs were residing long term. Scale bars represent 100  μm.

### 3.7 Deep Tissue Visualization

To demonstrate the capability of MPL for thick-tissue mapping, intact lung samples were imaged at depths exceeding 600  μm. This technique preserved the three-dimensional (3D) alveolar structure while revealing localized AuNPs (Fig. 6).

**Fig. 6:**
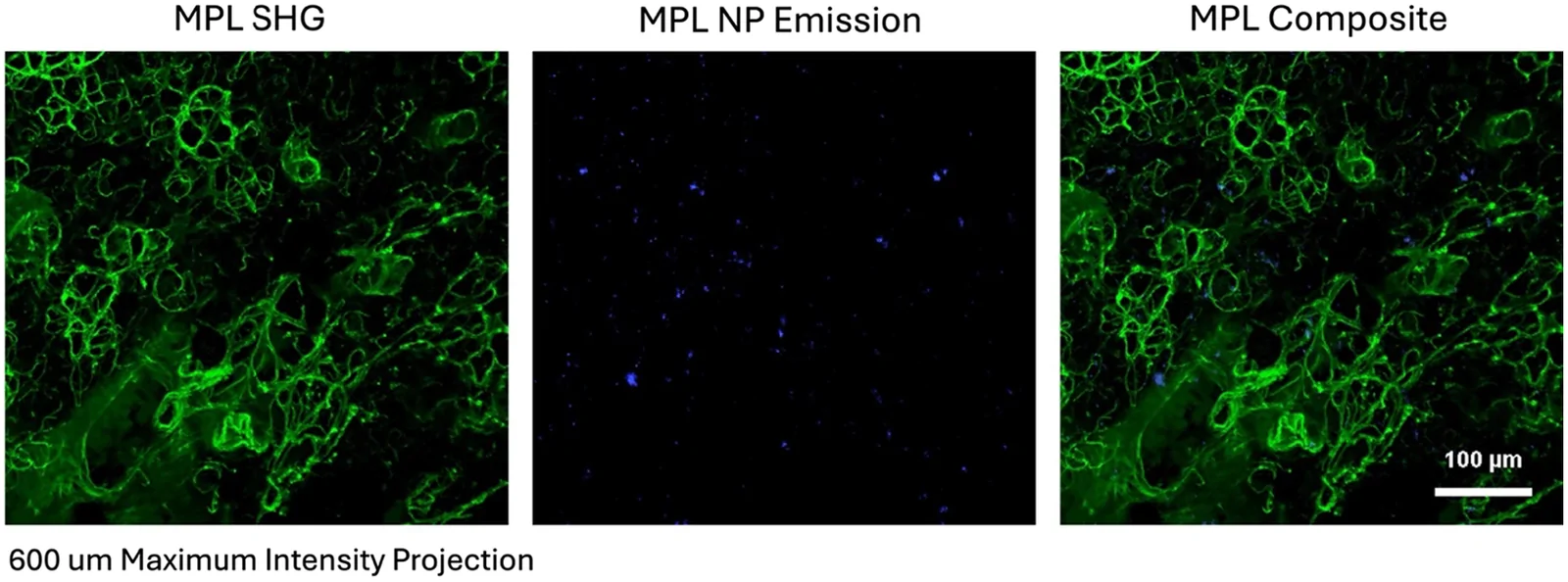
Deep-tissue MPL imaging of AuNPs in intact lung tissue. Maximum intensity projection of a z-stack acquired from intact lung tissue, showing >600-μm imaging depth. AuNPs are visualized in blue amidst the autofluorescent alveolar tissue background depicted in green.

The MPL imaging strategy demonstrated exceptional versatility, extending beyond surface-level observations to achieve high-resolution imaging at significant depths within uncleared, opaque tissue specimens. By bypassing the need for chemical clearing agents, we preserved the biological integrity of the sample, as evidenced by our successful visualization of AuNPs (highlighted in blue) through a 600-μm depth in fresh lung tissue (Fig. 6).

Beyond static depth profiling, our strategy enabled the capture of dynamic, volumetric data within uncleared specimens. We successfully generated a 3D reconstruction video of the 600-μm lung volume, providing a continuous perspective of the tissue architecture and NP distribution across various focal planes (SI Video 1). By scanning through successive depths, the video illustrates the seamless integration of the AuNP MPL within the wider cellular landscape. This capability highlighted the stability and penetration power of our imaging platform, allowing for a comprehensive spatial analysis of the lung’s internal environment without the need for physical sectioning.

## 4 Discussion

Our results demonstrated that inhaled 50 nm AuNPs remained localized in the lungs for up to 1 month without significant systemic translocation. The linear dose-response model observed at 1 week suggests that lung concentration can be predictably controlled in inhalation studies. Images at 1 month validate that the AuNPs are still detectable with MPL and highlight the importance of this developed label-free approach for long-term assessment. Our biodistribution findings are consistent with previous inhalation studies demonstrating limited systemic translocation of 50 nm AuNPs.^24^^,^^46^^,^^47^ Moreover, we demonstrated the ability to directly visualize retained gold nanoparticles across intact histological specimens without external labels while maintaining spatial tissue context.

Nonaerosolized intranasal administration was selected as a reproducible proof-of-concept pulmonary delivery method as it has been previously used in nanomedicine studies.^48^^,^^49^ Although aerosol inhalation devices or intratracheal methods offer alternative deposition profiles, liquid intranasal instillation allows direct, controlled dosing to the lower respiratory tract without requiring complex aerosol-generating equipment.^49^ Consequently, it serves as a common benchmarking approach to evaluate particle uptake, biocompatibility, and local lung distribution in nanomedicine and nanotoxicology studies.^50^ Future work will investigate aerosolized delivery systems to further establish translational relevance.

To navigate the complex environments within the lung, we utilized the intrinsic tissue autofluorescence as a natural, high-contrast cellular map. This structural framework allows us to precisely localize and overlay AuNPs, which are identified through their distinct MPL signals. This multi-channel approach ensures that the functional data from the nanoparticles is always contextualized within the native architecture of the deep tissue.

Using this multichannel approach, our pathologist confirmed that the AuNPs successfully reached the alveolar sacs, the primary sites for gas exchange, rather than remaining localized in the upper airways. Beyond the alveolar sacs, the AuNPs were also detected within the interstitial spaces and surrounding soft tissue. Evidence from MPL imaging further suggested that these NPs could be phagocytized by alveolar macrophages and could eventually drain toward the lymphatic system. This migration pattern could reveal higher gold concentrations within the lymph nodes over longer periods of time. Further investigation utilizing our label-free MPL imaging approach could validate this theory of long-term biodistribution and retention within the next draining lymph nodes from the lung.

Looking forward, the integration of the developed MPL imaging approach with conventional histopathological techniques offers a powerful framework for assessing the long-term biological and toxicity impact of AuNP retention in the lung. Future studies can utilize MPL imaging to provide precise, label-free localization of AuNPs within the intact pulmonary architecture while simultaneously employing specialized stains to evaluate tissue-level effects. For example, combining MPL with Masson’s Trichrome would allow researchers to correlate the physical presence of gold NPs with interstitial fibrosis or collagen deposition, while IHC staining for CD3 and TUNEL assays could visualize T cell-mediated inflammation and cellular apoptosis, respectively. By layering these high-resolution spatial maps of NP distribution over traditional morphological assessments, researchers can move toward a comprehensive understanding of long-term biocompatibility. The proposed multimodal approach has the potential to demonstrate whether long-term retention of AuNPs induces any overt pulmonary toxicity, significant immune activation, or widespread fibrosis across various doses and time points.

## 5 Conclusions

MPL imaging offers a robust, label-free alternative for tracking AuNPs in respiratory tissue, providing sensitivity that far exceeds the capabilities of traditional brightfield microscopy. This feasibility study demonstrates the successful application of an established label-free MPL platform to pulmonary nanotoxicology and inhalation biodistribution studies. Using MPL, we showed that 50 nm AuNPs administered intranasally remain strictly sequestered within the pulmonary system, with no evidence of systemic translocation to secondary organs over a one-month period. By enabling the visualization and quantification of dose-dependent aggregation within intact, deep tissue, MPL imaging confirms that AuNPs persist long-term while the surrounding pulmonary architecture remains preserved for morphological analysis. Ultimately, these findings indicate that the distribution and concentration of retained AuNPs can be predictably modeled in fixed histology sections, establishing a critical foundation for the use of MPL imaging in future toxicological assessments and the development of therapeutic inhalation strategies.

## 6 Appendix

Supplementary Material includes an experimental design schematic, large field of view MPL imaging, perfusion versus nonperfusion MPL imaging, MPL z-stack video (Video 1) of uncleared lung tissue with >600  μm. imaging depth. Raw data available upon reasonable request.

## Supplementary Material

10.1117/1.BIOS.3.4.043105.s01

10.1117/1.BIOS.3.4.043105.s1

## Data Availability

All data in support of the findings of this paper can be found either within the article or are available in our data repository at University of Southern California.
