# Supplementary material for: Feasibility of label-free multiphoton luminescence imaging to track the fate of inhaled gold nanoparticles in ex vivo lung tissue
Source: Biophotonics Discov. 2026 Sep 29;3(4):043105. doi: 10.1117/1.BIOS.3.4.043105 (PMC13622989; doi:10.1117/1.BIOS.3.4.043105)
Supplement: Supplementary file 1 [file BIOS_003_043105_SD001.pdf]

Figure S1. Experimental Design

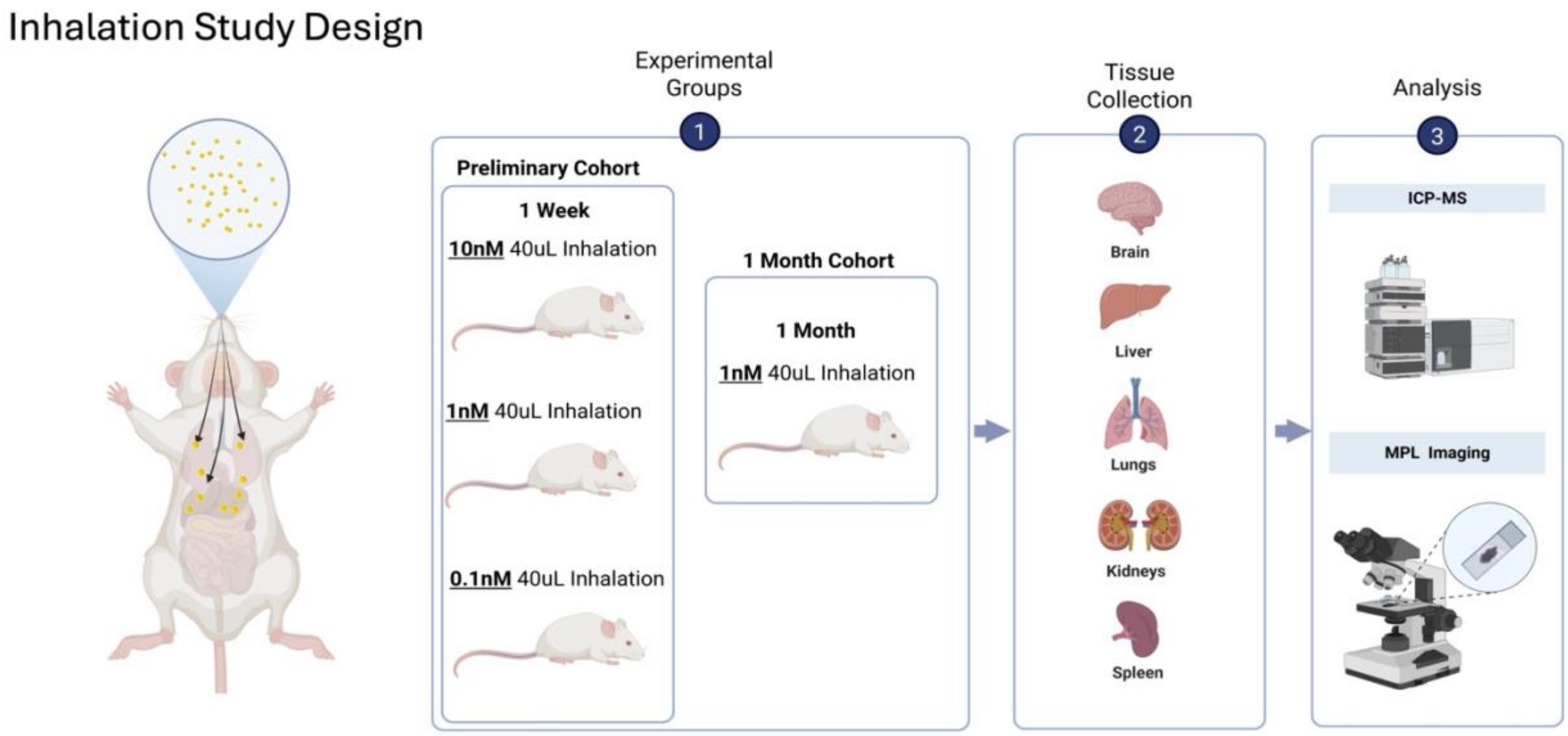

Figure S1. Experiential setup for inhalation study. Mice were dosed with various doses for the 1 week post inhalation time point, either 0.1 nM, 1 nM, or 10 nM of AuNPs via intranasal inhalation. Another group was looked at 1 month post inhalation of 1 nM AuNPs. Various organs were harvested for both ICP-MS and MPL imaging analysis.

Figure S2. Large FOV H&E Brightfield vs Multiphoton Luminescence Imaging

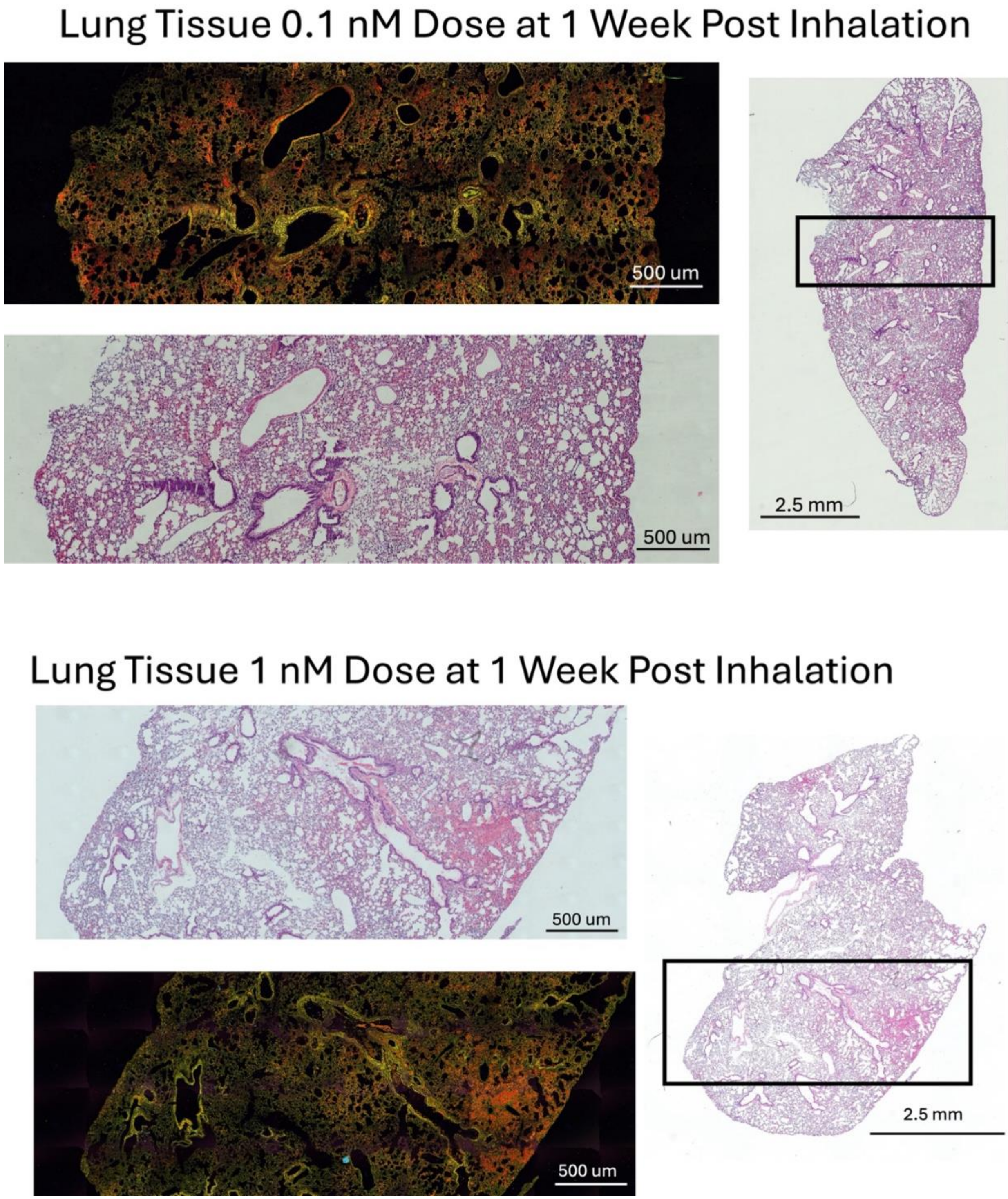

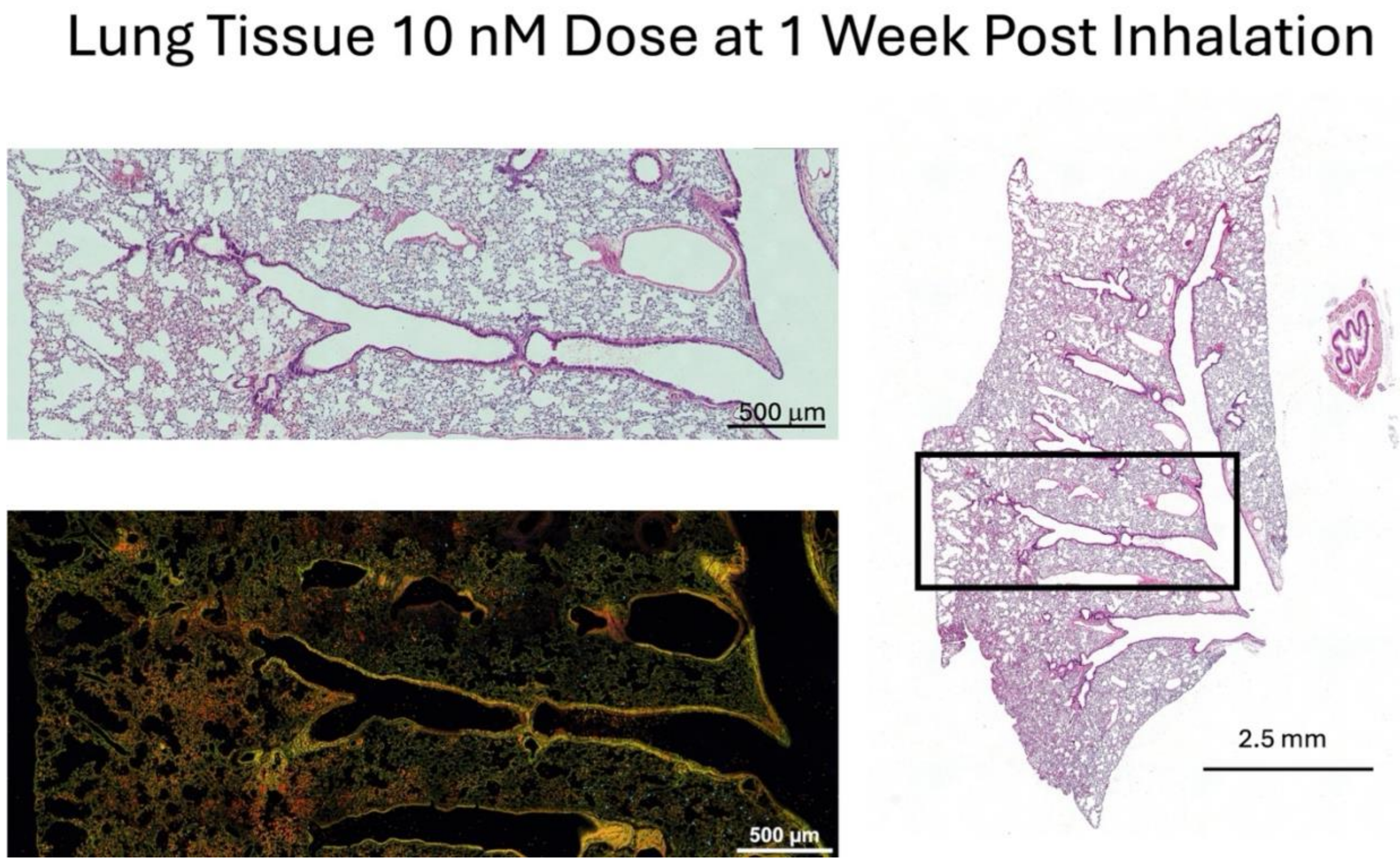

Figure S2. Large area brightfield and multiphoton luminescence (MPL) images of FFPE H/E stained mouse lung sections. Mice were dosed with either 0.1 nM, 1 nM, or 10 nM of gold NPs via intranasal inhalation. Notice how brightfield imaging is unable to identify the presence of the gold NPs across the histology section. MPL imaging clearly highlights the regions across the sample containing gold NPs. Gold NPs are revealed in blue. Zoomed in areas of MPL imaging are available in Figure 4. Notice how the cellular features of the alveoli are visually apparent and preserved in the multiphoton images. Eosin autofluorescence and tissue autofluorescence/second harmonic generation highlight alveoli and tissue structure in yellow/green.

Figure S3. Perfusion vs Non-Perfusion MPL Imaging

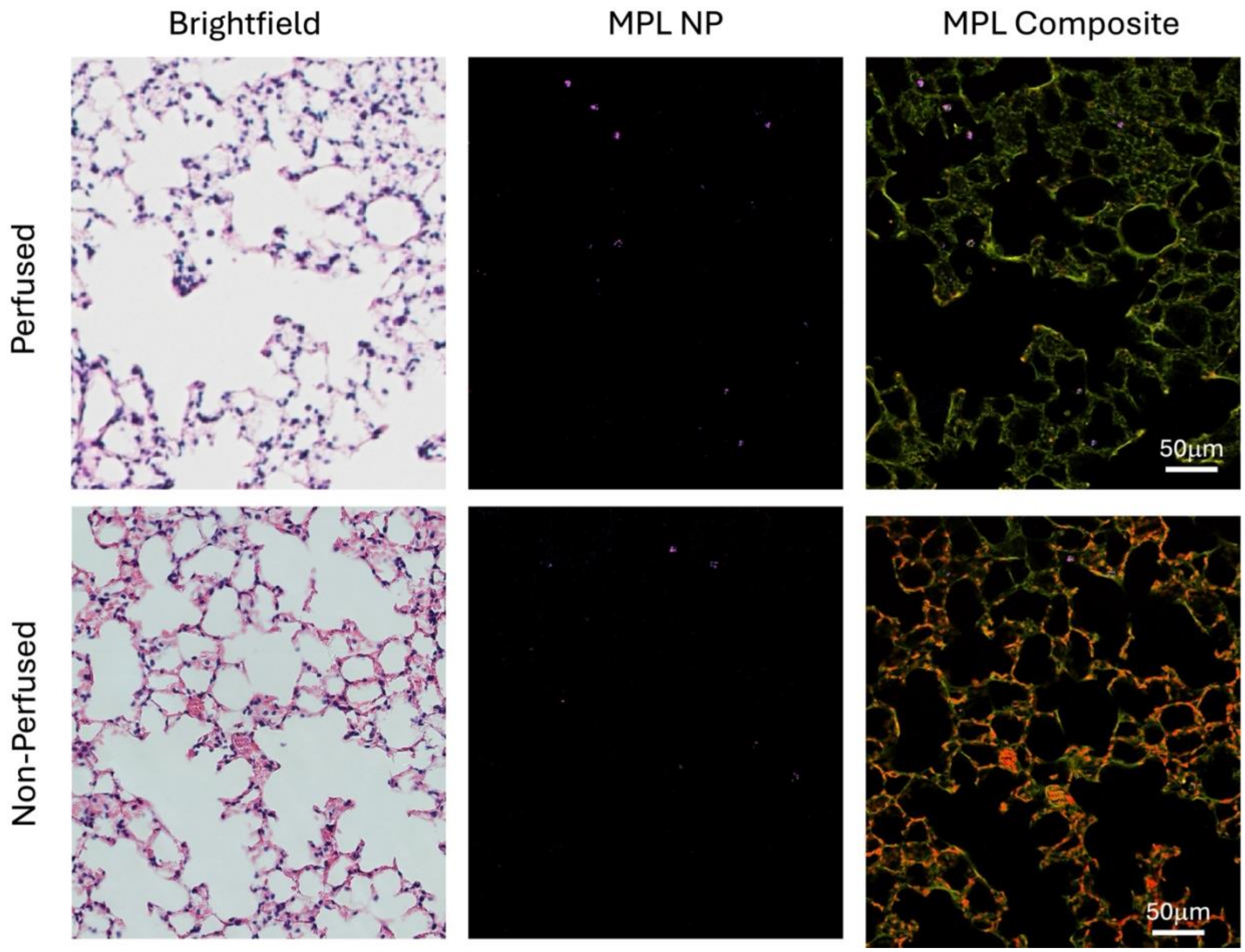

Figure S3. Brightfield and MPL imaging of lung tissue in perfused and non-perfused mice. Notice how the non-perfused lung tissue contains many red blood cells detectable across the alveoli structures in both the brightfield and MPL images. Although perfused images have less red blood cell autofluorescence contribution in the TRITC channel, the AuNPs are easily detectable in both groups. Perfusion does not appear to affect our ability to identify and localize AuNPs in lung histology tissues using MPL imaging. Scale bar 50 μm.

SI Video 1: Uncleared Lung with >600 μm Imaging Depth, 1 Week post Inhalation of 1nM Gold NPs

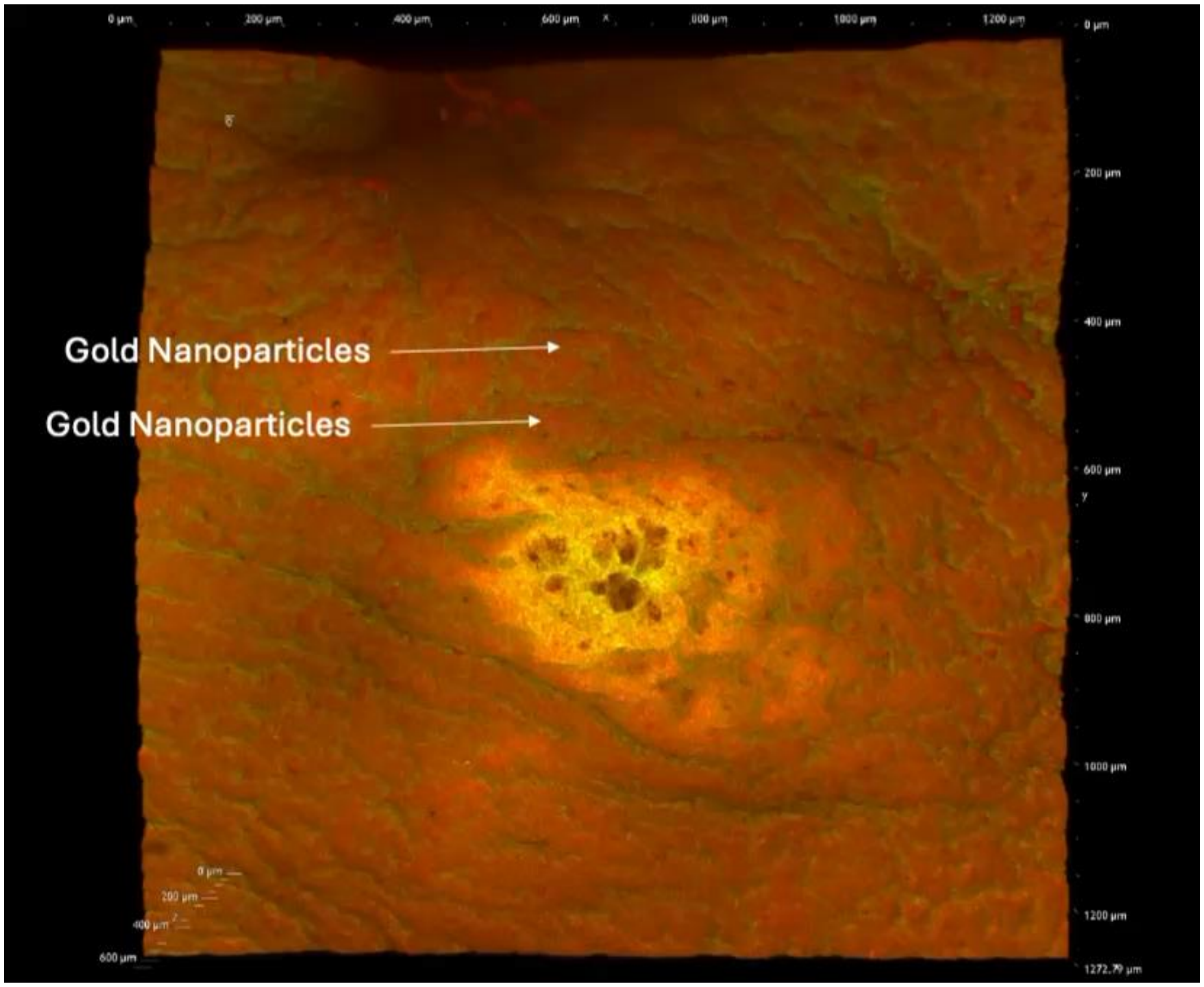

SI Video 1. This video depicts a z-stack of MPL images generated from freshly harvested lung tissue that has not been modified with clearing agents. Notice the AuNPs that come into view across the different depths over the 600 μm deep imaging field of view.
